# Development, Characterisation and Application of Monoclonal Antibodies for the Detection and Quantification of Infectious Salmon Anaemia Virus in Plasma Samples Using Luminex Bead Array Technology

**DOI:** 10.1371/journal.pone.0159155

**Published:** 2016-07-19

**Authors:** R. Hoare, K. D. Thompson, T. Herath, B. Collet, J. E. Bron, A. Adams

**Affiliations:** 1 Institute of Aquaculture, School of Natural Sciences, University of Stirling, Stirling, FK9 4LA, United Kingdom; 2 Moredun Research Institute, Pentlands Science Park, Penicuik EH26 0PZ, United Kingdom; 3 Department of Animal Production, Welfare and Veterinary Sciences, Harper Adams University, Newport, Shropshire TF10 8NB, United Kingdom; 4 Marine Scotland, Marine Laboratory, 375 Victoria Road, Aberdeen AB11 9PA, United Kingdom; Friedrich-Loeffler.Institut, GERMANY

## Abstract

Infectious salmon anaemia virus (ISAV) is an orthomyxovirus that has had a significant economic impact on Atlantic salmon farming in Europe, North America and Chile. Monoclonal antibodies (mAbs) were developed against Segment 3 (encoding the viral nucleoprotein, NP) of the virus. Six of the mAbs were shown to be specific to ISAV and recognised all isolates from Scotland, Norway and Canada. They reacted with ISAV in enzyme-linked immunosorbent assay (ELISA), indirect fluorescent antibody technique (IFAT) and western blotting. They were also used to develop a novel detection method based on Luminex (Bio-Plex) bead-based flow cytometric technology for the detection of ISAV in the plasma of Atlantic salmon (*Salmo salar* L.) smolts experimentally infected with ISAV. Fish were challenged by intraperitoneal (i.p.) injection of virus at 50% Tissue Culture Infective Dose (TCID_50_) = 2.8 x10^6^ per animal. Virus present in plasma of infected fish, collected at 0, 4, 8, 12, 16, 21 and 28 days post infection using a non-lethal sampling method (n = 12 at each time point), was quantified using the optimised Bio-Plex assay. The results obtained with this assay were compared with absolute quantification of the virus by RT-qPCR using SYBR Green I and TaqMan chemistries. The Bio-Plex assay developed using the NP mAbs appears to be a rapid, sensitive method for detecting and quantifying ISAV in small volumes of fish plasma and has the potential to be multiplexed for the detection of other fish pathogens (e.g. during co-infections). To our knowledge this is the first report of the use of Luminex (Bio-Plex) technology for the detection of a fish pathogen.

## Introduction

Infectious salmon anaemia (ISA) is a systemic infectious disease of farmed Atlantic salmon (*Salmo salar* L.), which has had a significant economic impact on salmon farming, in particular in Norway and Chile [1]. The causative agent of the disease, Infectious salmon anaemia virus (ISAV), is an enveloped, negative sense single stranded RNA virus of genus Isavirus, family Orthomyxoviridae [2]. ISAV is listed as a notifiable disease by the World Organisation for Animal Health [3]. The first cases of ISAV were reported in Norway in the 1980s [4] and cases have since been reported from Canada (1996, 1998, 2012), Scotland (1998), Faroe Islands (2000), USA (2001) and Chile (2007, 2013) [3,5]. Studies of epidemics have shown that the virus is transmitted from infected sites to neighbouring sites, with farm proximity and visits by well boat being risk factors for the spread of the disease [3,5,6]. The disease is characterised by lethargy, haemorrhagic eyes, pale gills and a distended abdomen in infected fish. Mortality levels are variable during ISA outbreaks and can be as low as 0.5–1.0% per day, but without intervention cumulative mortality in infected populations can reach as high as 90% [3], emphasising the need for early diagnosis to control the spread of the virus. The virus can be detected in infected fish using a combination of methods specified by OIE [3], including immunofluorescent techniques (IFAT), immunohistochemistry (IHC), quantitative real-time RT-PCR (RT-qPCR) or by virus isolation. Control of ISAV relies on accurate methods for early detection such that RT-qPCR is currently the standard method for surveillance of infection for reasons of availability, utility and diagnostic specificity [3]. Vaccination has been carried out in Norway, Canada and Chile, however complete protection has not been achieved with these vaccines to date [3], although a recently developed DNA vaccine has been shown to provide good protection in laboratory-based experiments [7].

Previous studies have used monoclonal antibodies (mAbs) against the haemagglutinin on the virion surface in IFAT and IHC for the diagnosis of ISAV infection [3,8]. Both methods can be subjective and are not quantitative, leaving RT-qPCR as the method of choice for a definitive diagnosis. While sensitive and specific, the use of RT-qPCR requires highly trained personnel, expensive reagents and is time-consuming.

Bio-Plex technology (BioRad based on Luminex xMAP technology) is a bead-based technology that is widely used in human health and is being developed for veterinary medicine [9] as it allows the detection and quantification of multiple analytes from relatively small sample volumes. Bio-plex technology, with its potential to serve as an alternative diagnostic tool to conventional methods currently used such as ELISA and RT-qPCR, has yet to be developed for use in aquaculture. It has the potential to use up to 100 colour-coded fluorescent bead sets, containing different ratios of two spectrally distinct fluorophores, making each bead set distinguishable by its fluorescent emission when excited by a laser. Each bead set can be conjugated with a unique protein, peptide, oligonucleotide or antibody (e.g. anti-ISAV monoclonal antibody (mAb)). Coupled beads are then incubated with a sample (e.g. plasma from ISAV infected fish) in a 96 well ELISA plate format, followed by incubation with a biotinylated antibody (e.g. a different anti-ISA mAb) using streptavidin-phycoerythrin as the reporter. The assay is then read on a Bio-Plex reader, which contains a dual laser system that excites the fluorophores within the beads at one wavelength (classification laser) and the detection antibodies bound to the beads at a second wavelength (detection/reporter laser). As each bead flows through the reader, the internal fluorescent emission from the bead identifies which antibody is attached to the bead and at the same time the fluorescent emission from the detection antibody attached to the surface of the bead allows the amount of target bound to the antibody to be quantified. The mean fluorescence intensity (MFI) of the detection fluorescent antibody is obtained for 100 beads from each bead set.

The aim of the present study was to produce mAbs against ISAV, characterise these using ELISA, immunoblotting and indirect immunofluorescent antibody staining technique (IFAT), and then subsequently apply the mAbs in the development of a novel detection method based on Bio-Plex array technology to detect and quantify ISAV in the plasma of Atlantic salmon infected with ISAV. The Bio-Plex assay was validated by comparing the sensitivity and specificity of detection of ISAV in the serum samples with that of two RT-qPCR chemistries.

## Materials and Methods

### 2.1 Development of mAbs against ISAV

#### 2.1.1 Production of mAbs to ISAV

The work described was performed in accordance with the UK's Home Office use of animals in scientific procedures, regulated by the Animals (Scientific Procedures) Act 1986. All animal experiments were approved by the Ethics Committee of the Institute of Aquaculture, University of Stirling, UK. The mice used in the study were obtained from Charles River Laboratories (UK) and housed at the Animal Care Facility at the University of Stirling. All efforts were made to minimize suffering during procedures and the mice were sacrificed by Schedule 1 according to Home Office regulations (rising concentration of carbon dioxide) according to the Humane Killing of Animals under Schedule 1 to the Animals (Scientific Procedures) Act 1986. Twenty four Atlantic salmon were provided by Landcatch Natural Selection (Hendrix-Genetics) and transported to the Level 3 Biosecurity Aquarium Facility at Marine Scotland. At the termination of the experiment the fish were sacrificed by anaesthetic overdose (MS222) according to Schedule 1 methods.

Norwegian ISAV isolate (Glesvaer/400/90), kindly provided by Marine Science Scotland (MSS), Aberdeen, Scotland, UK, was used to produce the mAbs. The virus was cultured as described by Dannevig *et al*. [10] using preformed Atlantic salmon head kidney cells (SHK-1 cells established from a culture of Atlantic salmon head kidney macrophages as described by [11]) cultured in Leibovitz medium (L-15), supplemented with 5% foetal bovine serum (FBS, Australian batch), L-Glutamine (4 mM), 2 mercaptoethanol (40 μM), and penicillin/streptomycin (100 IU/ml/100 ug/ml) (Invitrogen). When cells showed 70–80% confluence, the medium was removed and cell culture supernatant infected with ISAV at a multiplicity of infection (MOI) of 0.1 in serum-free L-15, adsorbed onto the SHK-1 cells for a minimum of 4 h at 15°C, after which the flasks were re-supplemented with growth media and cells monitored every 2–3 days for the development of a cytopathic effect (CPE). Once full CPE had developed, the supernatant was collected and clarified by centrifuging at 2,500 x g for 15 min at 4°C. Antigen was purified from the supernatant using a caesium-chloride gradient [12], and emulsified with Titermax Gold adjuvant (Vaxcel^™^ Inc., USA) (50:50 v/v) to give a final viral protein concentration of 200 μg/ml.

Two 5 week old Balb/c female mice were immunised for mAb production. Samples of blood (0.5 mL) were taken from the tail veins of each mouse prior to immunisation and they were then immunised by intraperitoneal (i.p.) injection with 100 μl of emulsified ISAV (20 μg protein per injection). Four weeks later, the mice received a second i.p. immunisation. Ten days after the second immunisation, a sample of blood was collected from the tail vein of each mouse and the antibody titre against the virus determined by ELISA. The mouse with the highest antibody titre received a final boost of virus intravenously [20 μg protein in 100 μl of phosphate buffered saline (PBS:10 mM NaH_2_PO_4,_ 150 mM NaCl)], without adjuvant. Hybridoma production was performed using the spleen of this mouse 4 days later according to Neelam *et al*. [13]. The isotype of the mAbs was determined using a mAb isotyping kit (Sigma-Aldrich).

To assess the antibody titres before commencing fusion, test bleeds from the two mice immunised with ISAV were screened using an indirect ELISA [13]. The initial screening of the fusion was carried out by ELISA using cell culture supernatants (double screened against both infected and non-infected supernatants), while subsequent clonings were screened using CsCl-purified virus in the ELISA at a protein concentration of 10 μg/ml^1^ to coat the ELISA plates.

#### 2.1.2 Characterisation of the anti-ISAV mAbs

The ability of the mAbs to detect the virus in infected SHK-1 cells was examined by IFAT using cells grown on coverslips and infected with virus according to Dannevig *et al*. [10] and observed under a Leica TCS SP2 AOBS confocal laser scanning microscope. Immunoblotting was performed to characterise the mAbs and establish which viral proteins they recognised using CsCl-purified virus at 1 mg/ml on the gel [12].

#### 2.1.3 Specificity of mAbs

The mAbs were tested against a variety of ISAV isolates (kindly provided by MSS), shown in Table 1, by ELISA [8]. The specificity of the mAbs was tested against a variety of viral and bacterial fish pathogens: Betanodavirus; Infectious pancreatic necrosis virus; Salmon pancreatic disease virus; Infectious salmon anaemia virus; Sleeping disease virus; *Aeromonas salmonicida; Vibrio vulnificus; Vibrio harveyii; Vibrio anguillarum*; *Vibrio parahaemolyticus; Vibrio splendidus; Vibrio alginolyticus; Photobacterium damselae* subspecies *piscicida; Yesinia ruckeria; Mycobacterium fortuitum*; *Mycobacterium chelonae; Piscirickettsia salmonis* LF-89.

**Table 1:** Cross-reactivity of the anti- infectious salmon anaemia virus (ISAV) monoclonal antibodies with other ISAV isolates determined by an enzyme-linked immunosorbent assay.

mAb	CAN NBISA V01	SHK-1	NOR ILA8	NOR ILA10	NOR ILA36	SCOT 388/98	SCOT 390/98	SCOT 832/98	SCOT L. Nevis 90/98	NOR G1Esvr	NOR Glesvar /400/90
1	2.097	-	1.043	0.678	1.105	1.550	0.858	1.257	2.312	1.484	0.475
3	2.053	-	1.22	0.903	1.162	1.19	1.041	1.417	2.176	1.178	1.23
15	2.014	-	1.124	0.655	1.075	1.056	1.129	1.191	2.163	1.19	1.357
32	2.17	-	1.138	0.584	1.092	1.053	0.951	1.141	2.151	1.005	0.549
33	2.135	-	1.227	0.831	1.268	1.126	1.115	1.329	2.115	1.394	1.441
37	2.087	-	1.549	0.944	1.352	1.371	1.283	1.651	2.267	1.428	1.597
40	-	-	-	-	-	-	-	-	0.746	-	0.677
PBS	0.231	0.200	0.184	0.237	0.152	0.151	0.272	0.216	0.231	0.184	0.086

The positive threshold used for the assay was absorbance values (450 nm) two times that of the PBS negative controls. Values represent the mean value for three replicate wells

### 2.2 Development of Bioplex Assay for detection of ISAV

mAbs (aISAV mAb 3 and aISAV mAb 32) were tested by sandwich ELISA according to [14] to determine which to use as capture and detection in the Bio-Plex assay and to optimise the concentration to use for coupling to the beads. These mAbs were selected to use in the Bio-Plex assay as they have also been used to develop a lateral flow immunoassay for ISAV, the diagnostic sensitivity and specificity of which was reported previously [15].

#### 2.2.1 Biotinylation of detection mAb

Anti-ISAV mAb32 was biotinylated using the EZ-Link Sulfo-NHS-Biotin Kit (Thermo Scientific) according to the manufacturer’s instructions.

#### 2.2.2 Coupling of mAbs to magnetic microsphere beads

Anti-ISAV mAb 3 was coupled to a magnetic microsphere bead set (BioRad Laboratories, Luminex Corporation) using a 2-step carbodimide reaction. Beads were protected from light at all times. The beads (1.25 x 10^6^ beads) were activated with 1-ethyl-1-3-dimethylaminopropyl- carbodimide hydrochloride (EDC) and N-hydroxysulfosuccinimide (S-NHS) in 80 μl activation buffer (10 mM NaH_2_PO_4_) for 20 min at 22°C with rotation. The activated beads were pelleted using a magnetic separator (DYNAL) and the supernatant discarded. The antibody was sonicated for 2 min in a water bath prior to diluting it with coupling buffer (50 mM MES potassium salt) and then 500 μl of diluted antibody (10 μg/ml) were added to the pelleted beads and incubated with rotation for 2 h at 22°C. The beads were pelleted as before and washed twice with 500 μl PBS, re-suspended in antibody buffer (PBS/1% Bovine Serum Albumin/0.05% sodium azide) and stored in the dark at 4°C.

Validation of the coupling was performed by placing 500 μl of the bead suspension, containing 5000 coupled beads into two 1.5 ml Eppendorf tubes. Fifty μl of anti-mouse PE at 2 μg/ml was added to one of the tubes (positive tube) and both tubes were then incubated for 30 min on a rotator at 22°C. The tubes were placed on the magnetic separator and the supernatant was carefully discarded by pipetting. The beads were washed once with PBS, re-suspended in 125 μl of antibody buffer, transferred to a microtitre plate and analysed on the Luminex. A reading of greater than 2000 MFI in the positive tube signified successful coupling.

#### 2.2.3 Detection of virus in infected tissue culture supernatants and plasma samples from infected fish using the BioPlex assay

A standard curve for the Bio-Plex assay was prepared using ISAV Nevis strain SCO 390/98, cultured as described above in Section 2.1. The virus concentration present in harvested supernatants was 2.2 x10^6^ TCID_50_/ml. Dilutions of this supernatant were prepared (1/10, 1/25, 1/50, 1/100, 1/500, 1/1000 and 1/5000) in antibody buffer (PBS/1% BSA) for generation of a standard curve. Negative controls consisted of a pool of plasma samples from uninfected fish while positive control plasma (kindly supplied by Marine Scotland) was a pool of infected plasma obtained from a previous challenge with ISAV Nevis strain SCO 390/98 and confirmed by virus isolation on SHK-1 cells. To validate the assay, plasma from Atlantic salmon experimentally infected with ISAV as previously described under UK home office regulations [16] was tested. Immediately before injection (day 0), blood samples were collected using a non-lethal sampling method [16]. Briefly, to minimise stress in-tank anaesthesia was carried out. The water was slowly drained to 500 L and 400 mL of MS222 (Sigma) at 50 mg/L in tap water was poured into the tank through the automatic feeder opening. After 2 min the animals were sufficiently sedated to allow sample collection and returned into a tank with fresh aerated seawater for recovery. The sampling for the 12 fish lasted less than 7 min in total. The blood was withdrawn from the caudal vein, in the sagittal plane with a 1 mL syringe (Beckman Dickinson) attached to a gauge 23 needle (BD). Blood samples were subsequently collected at 4, 8, 12, 16, 21 and 28 days post infection (d.p.i.). Blood samples were centrifuged for 30 sec at 13,000 x g at 22°C, and the plasma was collected and stored at -80°C until analysed.

Optimal concentration of capture antibody coupled to the magnetic beads was determined by testing two concentrations (5, 10 μg/ml) and the optimal concentration of biotinylated detection antibody (10, 20 μg/ml) was similarly determined. Bead number per well (3000, 5000) was also optimised. In order to determine the optimal incubation conditions for the assay varying time and temperature of incubation with virus/plasma were tested (overnight at 4°C or 2 h at 22°C).

The optimised assay conditions were as follows: conjugated beads (anti-ISA mAb 3: 10 μg/ml, 5000 beads/well) were vortexed for 30 s to disperse the beads and 50 μl of the bead suspension were added to each well of a black 96 well ELISA plate (Greiner). The plate was placed on a 96 well Bio-Rad hand held magnetic washer for 1 min, and the buffer was removed from the wells by inversion of the plate while attached to the magnet washer. The dilutions of infected virus supernatant or plasma from fish infected with ISAV, diluted 1:2 in antibody buffer, was added to the wells of the ELISA plate in triplicate (50 μl/well) and incubated for 2 h. The supernatant was removed by magnetic separation as described above and microspheres were washed twice in antibody buffer (100 μl/well). Anti-ISA mAb 32-biotinylated was added to each well (20 μg/ml, 50 μl/well) and the plate was incubated for 1 h. The plate was washed twice as described above and incubated with streptavidin-PE at 20 μg/ml (50 μl/well) for 30 min. All incubations of the plate were performed on a plate shaker (IKA) at 800 rpm (30 s), followed by 300 rpm at 22°C in the dark. The plate was washed as above, microspheres were re-suspended in 125 μl/well of antibody buffer and analysed on the Luminex (100 beads per region). Results are reported as the mean fluorescence intensity (MFI) of 100 counted beads.

### 2.3 Quantification of virus by absolute qRT-PCR

Quantification of virus by absolute qRT-PCR was carried out according to the method of Workenhe *et al*. [17] and Fronhoffs *et*. *al*. [18] with modifications. Viral RNA was extracted from the 1/10 dilution of virus used for the BioPlex standard curve or plasma sampled from infected fish, using a Nucleospin^®^ RNA Virus kit (Macherey-Nagel) according to the manufacturer’s instructions. The RNA was stored at -70°C until reverse transcribed to construct cDNA using a high-capacity cDNA Reverse Transcription kit (Applied Biosystems, USA).

To obtain a large amount of *in-vitro* transcribed ‘sense’ RNA (IVT RNA) for use in downstream applications, a linearized DNA template was obtained using a conventional RT-PCR. For the PCR, a primer pair targeting segment 8 [19] of the virus by tagging a T7 RNA polymerase promoter to the 5’ end of the forward primer (5’-TAATACGACTCACTATAGGGCTACAC AGCAGGATGCAGATG-3’) was annealed with normal reverse primer sequence (5’CAGGATGCCGGAAGTCGAT-3) [18]. The 100 μl of reaction mixture contained 15 μl cDNA, 5 μl modified forward primer and 5 μl reverse primer (10 pM/ml), 25 μl nuclease-free water and 50 μl myTaq^™^ PCR Master mix (Thermo Scientific, UK). The thermal cycle conditions consisted of 95°C initial denaturing for 1 min, followed by 39 cycles of 15 s denaturing at 95°C, 15 s annealing at 60°C and 10 s extension at 72°C. The PCR product was then purified using a Promega PCR Clean-up kit (Invitrogen, UK). The DNA content of the purified PCR product was measured on a Nanodrop^®^ 1000 and assessed on a 1% agarose gel before being used as the template to obtain IVT RNA using a MEGAscript^®^ high yield transcription kit (Ambion, UK) according to manufactures instructions. The reaction mix was purified using phenol:chloroform as recommended by the manufacturer to clean short transcripts. The RNA content and the size of the purified product was assessed using Nanodrop^®^ 1000 and 1% agarose gel respectively, before 1μg/μl of IVT RNA per reaction was reverse transcribed to construct cDNA using a High-capacity cDNA Reverse Transcription kit (Applied Biosystems, USA) for the subsequent molecular applications.

#### 2.3.1 qRT-PCR with SYBR Green I chemistry or TaqMan^®^ chemistry

Real time PCR was performed on first strand cDNA (reverse transcribed from *in-vitro* transcribed RNA) using the Eppendorf^®^ RealPlex^2^ Mastercycler gradient S instrument, using ISAV Segment 8 primer pair ISAV8F/ISAV8R (amplifying a 104 bp product), [19], either with SYBR^®^ Green I (Thermo Scientific) master mix or with TaqMan^®^ master mix. For the SYBR green PCR assay, the 20 μl PCR mix reaction mix was comprised of 5 μl of cDNA and 15 μl of master mix prepared using 1 μl of the 10 pmol forward and reverse primers, 10 μl SYBR^®^ Green I and 3 μl of nuclease free water. The cycling conditions consisted of 95°C initial denaturing for 15 s, followed by 40 cycles of 15 s denaturing at 95°C, 30 s annealing at 60°C and 30 s extension at 72°C. The melting curve analysis was performed from 60°C to 95°C in 0.1°C/s increments to assess the specificity of the RT-PCR products.

The PCR mix for the Taqman assay consisted of 5 μl of cDNA dilution and 15 μl of master mix prepared using 1 μl of a 20X TaqMan probe/primer mix (5 μM probe: FAM-labelled ISAV segment 8 and 18 μM each primers) [19], 10 μl TaqMan master mix (Applied Biosystems) and 4 μl of nuclease free water. The PCR amplification consisted of 1 cycle of enzyme activation for 2 min at 50°C followed by denaturation at 95°C for 20 s, and 40 cycles of denaturation at 95°C for 3 s, 30 s annealing at 60°C.

For generation of the standard curve, the copy number of the *in-vitro* transcribed RNA was calculated as described by Fronhoffs [18]. Serial 10-fold dilutions of the cDNA transcripts from IVT RNA were prepared in nuclease free water starting with highest concentration of 1.7 x 10^8^ copies/μl. For both SYBR green and Taqman assay, the Ct values of the serial dilutions of cDNA obtained from IVT RNA were used to generate a standard curve plot of cycle number versus log concentration in the *realplex* software V2.2 (Eppendorf). The quality of the standard curve was judged by the slope of the curve and the correlation coefficient (r). The slope of the line was used to estimate the estimate the efficiency of the target amplification using the equation E = (10^−1/slope^)-1.

### 2.4 Data Analysis

Diluted virus culture supernatant and plasma samples (n = 24, 7 times points) were assayed in triplicate on the Bio-Plex. The limit of detection (LOD), defined as the lowest concentration of analyte that can be detected, and the limit of quantification (LOQ), defined as the lowest concentration of analyte that can be quantified, were determined by measuring 17 wells containing assay buffer alone. To quantify LOD, 3 standard deviations (S.D.) were added to the mean fluorescence intensity (MFI) of the blank wells and for the LOQ, 6 S.D. were added to the MFI of blank wells and concentration was determined from the standard curve. The Bio-Plex assay data were analysed using linear regression curves to determine concentration of analyte following data acquisition using the BioRad Manager software 4.1.1. The MFI values were transformed to the log_10_ scale improving the approximation of the data to the normal distribution.

## Results

### 3.1 Characterisation of Monoclonal Antibodies

After three rounds of cloning, 7 clones were obtained that recognised only the virus and did not react with SHK-1 cells. Of these, only six mAbs (mAbs 1, 3, 15, 32, 33 and 37) recognised all ISAV isolates tested representing Canadian, Scottish and Norwegian isolates (Table 1). None of the mAbs reacted with the other bacterial and viral fish pathogens screened by ELISA (data not shown).

**Fig 1:**
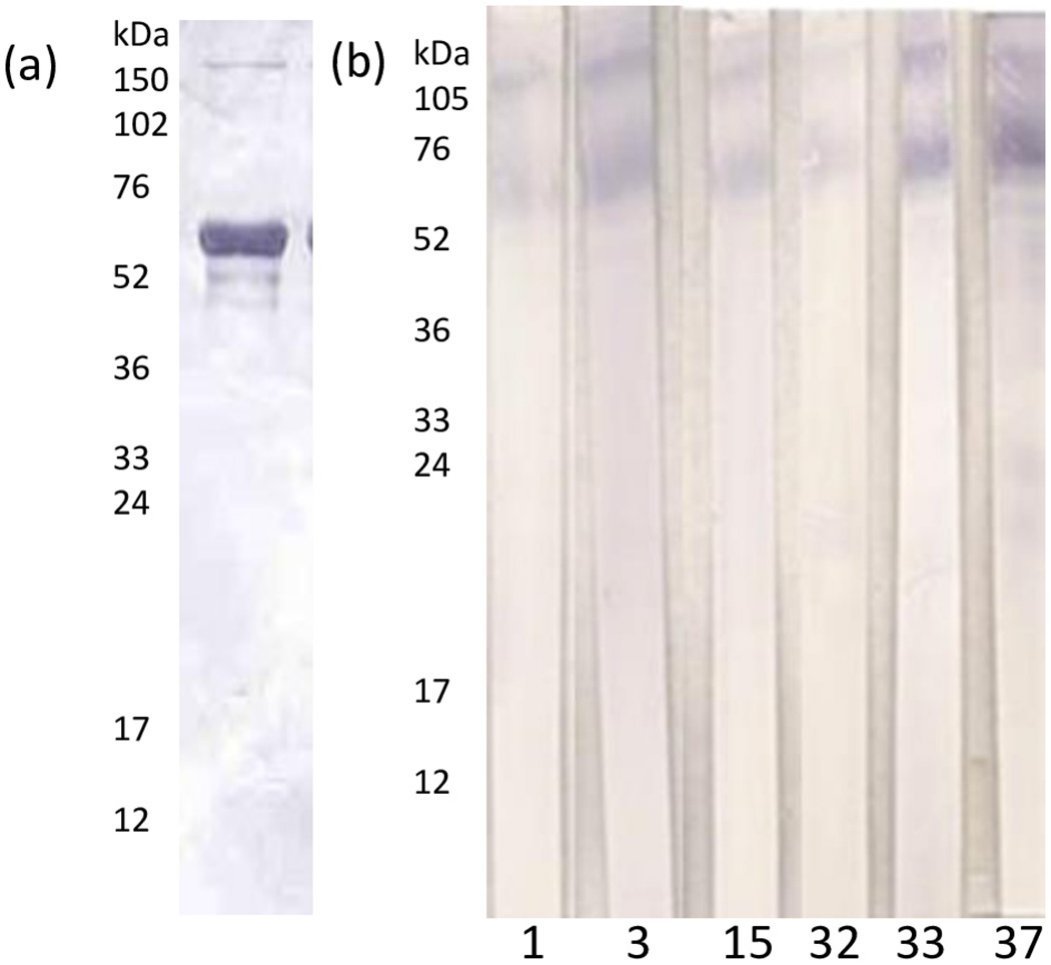
Western blot analysis using caesium chloride purified anti-infectious salmon anaemia virus (ISAV) (Norwegian isolate Glesvaer/400/90). (a) with polyclonal serum from the mouse used for the fusion (b) anti ISAV monoclonal antibodies (Mabs 1,3,15, 32, 33, 37) which gave a positive reaction in Western blotting.

**Fig 2:**
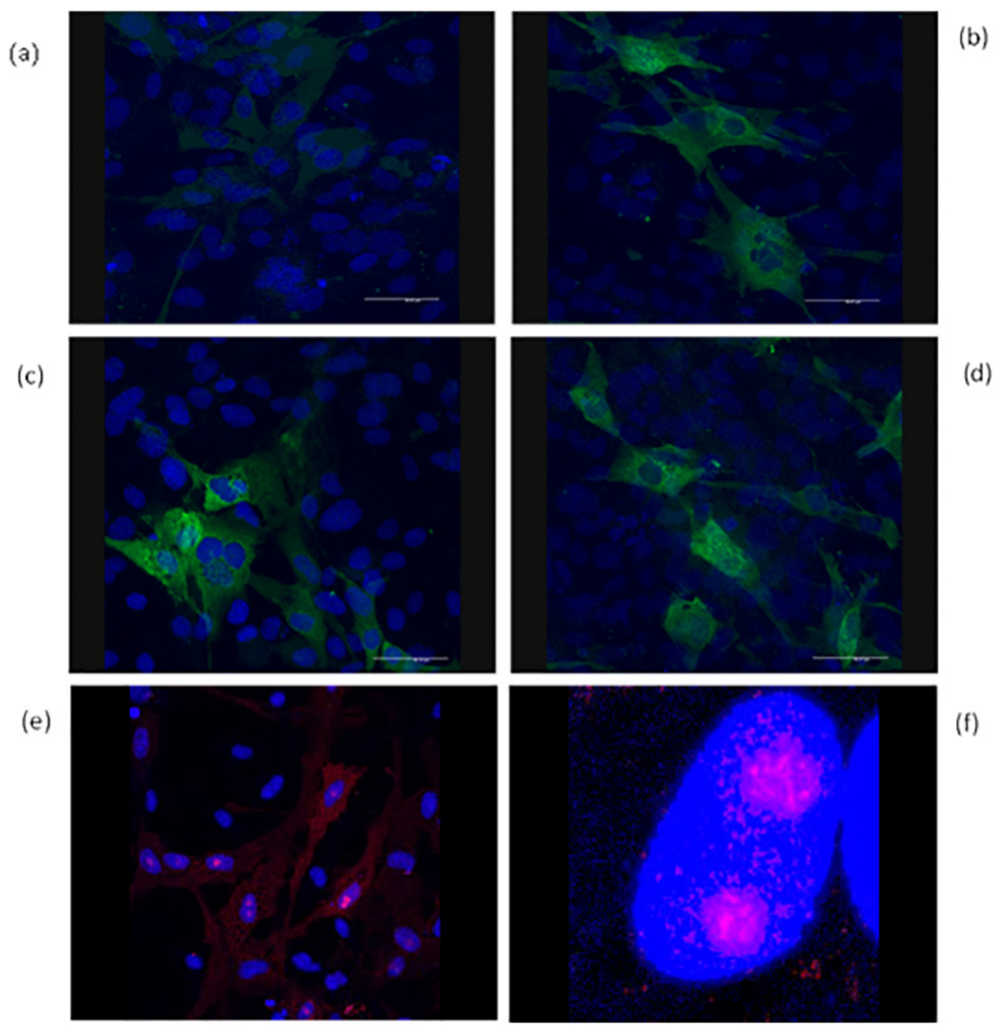
Characterisation of mAbs by immunocytochemistry. Confocal imaging of SHK-1 cells infected with ISAV (Norwegian isolate Glesvaer/400/90) for 3 days, then stained in immunocytochemistry with (a) mAb 15 against non-infected SHK-1 cells (i.e. no cross-reaction of MAb with host cells) (b) mAb 15; (c) stained with mAb 33; (d) stained with mAb 37; (e and f) stained with mAb 32. (Red and green represent antibody staining and blue represents nuclear staining with DAPI).

The mAbs were characterised by immunoblotting to establish which viral proteins they recognised. The six mAbs that reacted with all the ISAV isolates tested in ELISA (mAbs 1, 3, 15, 32, 33 and 37), recognised the nucleoprotein (NP) of the virus, represented by the band present at 66–71 kDa in immunoblotting (Fig 1). All of these mAbs detected the virus in infected SHK-1 cells, by IFAT using cells grown on coverslips and infected with virus and examined by confocal microscopy. The virus could be clearly seen in the SHK-1 infected cells, as shown in Fig 2 for mAbs 15, 32, 33 and 37. There was very little back-ground fluorescence with non-infected cells used as a negative control (Fig 2a). As well as seeing stained virus in the cytoplasm of the cells, there was also strong staining within the nucleus of the cells, especially within the nucleolus, shown in Fig 2e and 2f with mAb 32.

### 3.2 Development of Bio-Plex assay for detection of ISAV

#### 3.2.1 Optimisation of standard curve for detection of ISAV- Bio-Plex

Detection of cultured virus by ELISA was optimal using aISAV mAb 3 at 5 μg/ml (S1 Fig) as capture. Subsequent detection of virus in infected plasma showed marginally higher OD when aISAV mAb 3 was used at 10 μg/ml (not shown). This concentration of aISAV mAb 3 was then used for coupling to the BioPlex beads and the detection antibody concentration (anti-ISAv mAb 32-biotinylated) was optimised at 20 μg/ml.

**Fig 3:**
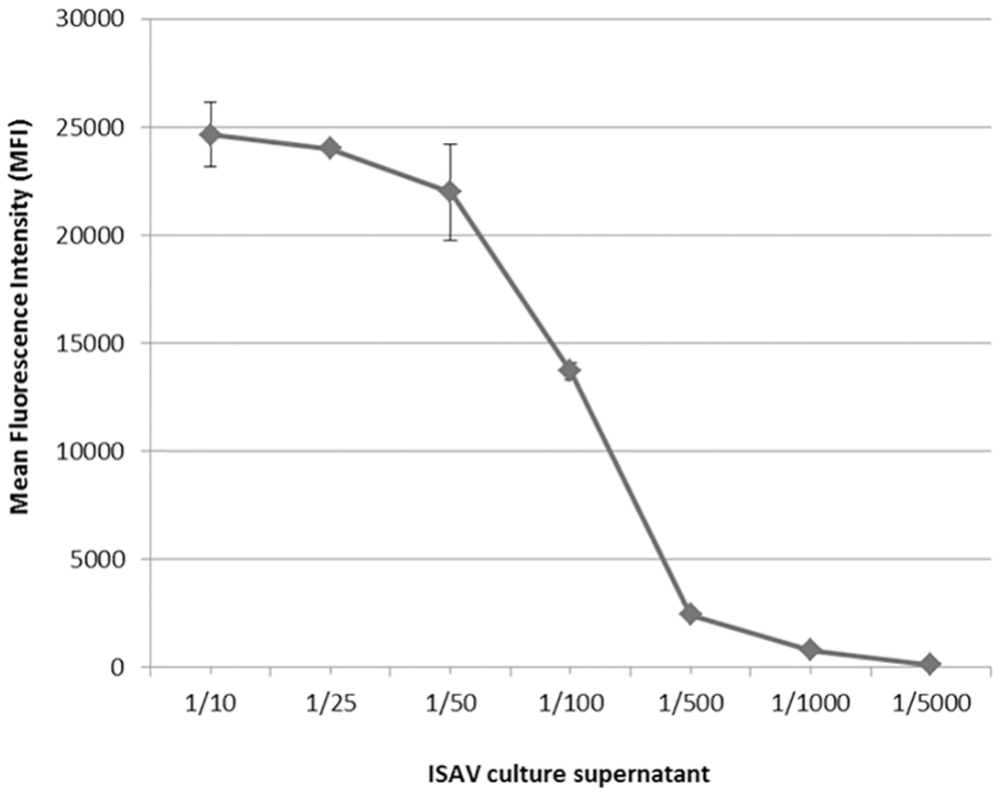
Construction of standard curve to detect ISAV using Bio-Plex. Standard curve of infectious salmon anaemia virus (ISAV) detection with a BioPlex sandwich immunoassay using cell supernatants from SHK-1 cells infected with ISAV Nevis strain SCO 390/98 (diluted from 1/10 to 1/5000) with 5000 beads/well.

A standard curve was constructed for the detection of ISAV by Bio-Plex (Fig 3). The conjugated beads (anti-ISAV mAb3) were incubated with culture supernatant of infected SHK-1 cells, serially diluted to produce a standard curve for dilutions from 1/10 to 1/5000. Increased sensitivity was obtained when 5000 beads/well were used compared with 3000 beads/well. When different incubation times with the virus were performed *i*.*e*. overnight at 4°C and 2 h at 22°C, there was no loss of sensitivity using a shorter incubation time. The endpoint titre of the assay was 1:5000 (99% confidence level) [20]. The LOD of the optimised assay was MFI: 303.73 and the limit of quantification (LOQ) was MFI: 374.99, equivalent to a dilution of cultured ISAV between 1/1000 (MFI 801.6) and 1/5000 (MFI 137.3).

#### 3.2.2 Detection of ISAV in challenged fish by Bio-Plex

**Fig 4:**
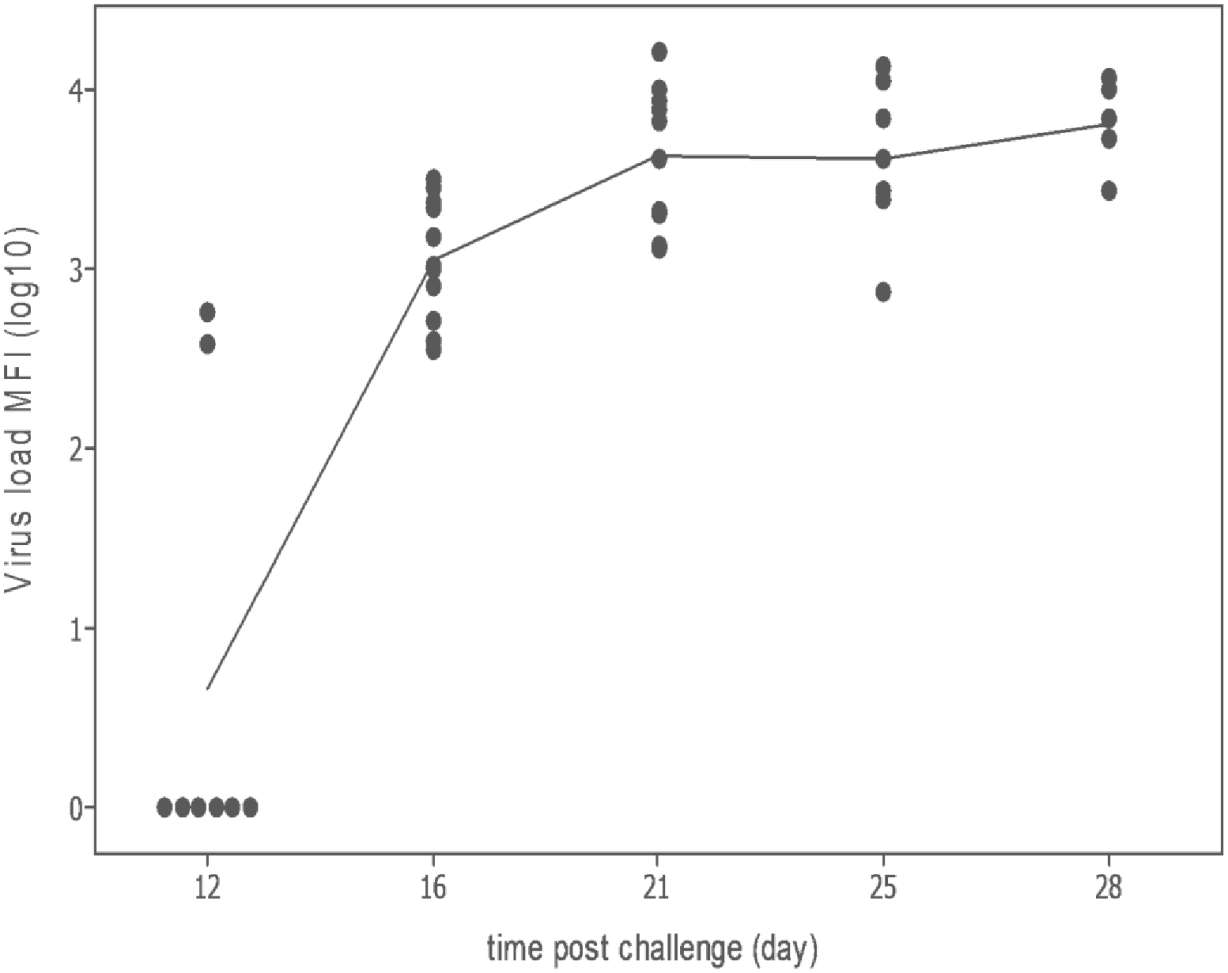
Validation of Bio-Plex assay using infected plasma. Detection of infectious salmon anaemia virus (ISAV) in the plasma of individual fish infected with the virus using a BioPlex Assay. Blood was non-lethally sampled from ten fish on 0, 4, 8, 12, 16, 21 and 25 days post-injection. The mean fluorescence intensity (MFI log_10_) of each fish measured BioPlex assay is plotted against time post-challenge.

Bio-Plex detection of virus in the plasma of infected fish was first observed for samples taken at 12 d.p.i., with 2/11 fish testing positive for ISAV antigen (mean MFI 478.4 ± 132.37, equivalent to a titre of between 1000 and 5000). By the end of the experiment (28 d.p.i.) 5/5 of fish tested positive with a mean MFI of 7052.78 ± 837.67 (titre 887.26 ± 433.63) with a range of 464.39 to 1544.42. No virus was detected in any of the control fish. A plot of virus detection (using the Bio-Plex) versus d.p.i. (MFI log_10_) showed a plateau in viraemia at 21 d.p.i. (Fig 4). The range of the virus titre detected with the Bio-Plex over the course of the infection was 217.31 to 3,816.6. Inter-assay variability was evaluated by measuring the same positive control sample on five different days, with low a Coefficient of Variation (CV) of 7.6% obtained. Intra-assay variability (multiple replicates of positive control sample measured on the same day) was also low with a CV value of 9.1%.

The detection of the virus was compared by titrating uninfected and infected plasma by ELISA and BioPlex methods (S2 Fig). The BioPlex assay proved more sensitive, titering to 1:128 whereas by ELISA the titer was 1:16. The background was relatively higher in the ELISA assay which gave a higher cut-off for detection of the virus (LOQ = blk + 6xSD). Both assays proved specific for the virus as no signal was detected in negative plasma.

### 3.3 Detection of ISAV: Absolute quantification PCR

The RT-qPCR platform, targeting Segment 8 of the ISAV genome, was performed using SYBR^®^ Green real time chemistry and TaqMan^®^ chemistry. The assays were used to measure genomic viral RNA copy number present in virus absorbed SHK-1 tissue culture supernatant and plasma of experimentally infected fish and correlated with the newly developed Bio-Plex assay.

#### 3.3.1 Absolute qPCR: SYBR^®^ Green and TaqMan assays

**Fig 5:**
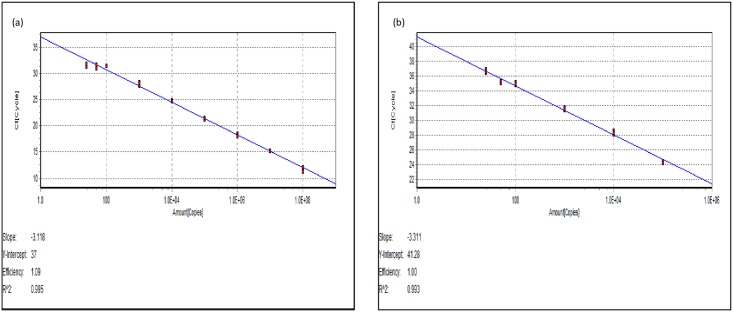
RT-qPCR standard curves. Standard curve relating viral copy number to Ct value from RT-Polymerase chain reaction using 10-fold dilutions of *in-vitro* transcribed RNA using (a) SYBR^®^ Green I and (b) TaqMan chemistries.

The dynamic range of the SYBR^®^ Green assay was 1.7 x 10^8^–50 copies/ μl and the detection limit was 50 RNA copy no (Fig 5a). The standard curve had an amplification efficiency of 1.09.

Similar to the SYBR^®^ Green assay, a 10-fold dilution series of cDNA was used to produce the standard curve for the TaqMan^®^ assay. The dynamic range of the TaqMan^®^ assay was 1.7 x 10^8^–25 copies/ μl and the detection limit was 25 RNA copies (Fig 5b). The standard curve had an amplification efficiency of 1.0.

#### 3.3.2 Correlation of detection of ISAV by qPCR and with the Bio-Plex assay

**Fig 6:**
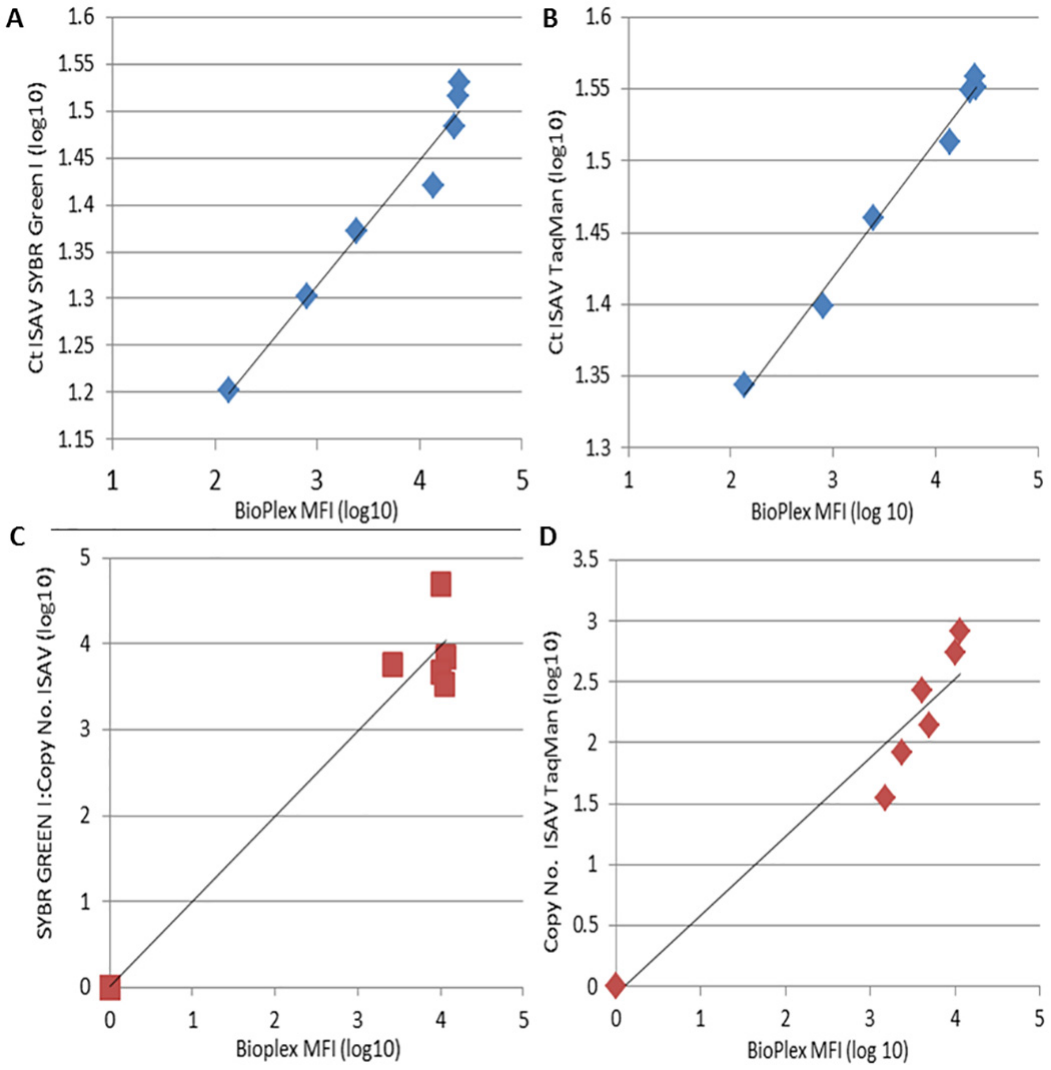
Correlation of detection of virus by Bio-Plex and RT-qPCR. Correlation between qPCR (Ct value) and Bio-Plex (MFI) detection of *in-vitro* grown ISAV standard curve by (A) SYBR GREEN I and (B)TaqMan; Correlation of detection of copy number of ISAV in plasma samples by (C) SYBR Green I (n = 13) and (D) TaqMan chemistries (n = 8).

The standard curve plots of Ct *versus* MFI for the SYBR^®^ Green I (Fig 6a; R = 0.979) and TaqMan chemistries are shown (Fig 6b; R = 0.994). There was a strong correlation between detection of ISAV with the Bio-Plex assay and with the standard curve for both qPCR assays and also detection of virus in infected/non-infected plasma samples (Fig 6c; SYBR Green I R = 0.989 5 fish/13 positive; Fig 6d; TaqMan R = 0.973, 6 fish/13 positive). Samples from the same fish taken at 16 and 21 d.p.i. were found to be positive by Bio-Plex and TaqMan qPCR, but were negative by SYBR Green I qPCR, revealing better sensitivity of the Bio-Plex assay and TaqMan qPCR (25 RNA copies) compared to the SYBR Green I assay (50 RNA copies). The plasma of one fish, measured at 12 d.p.i., was positive by Bio-Plex and negative by both qPCR methods.

### 3.4 Discussion

Monoclonal antibodies have proven to be useful tools in the diagnosis of fish diseases [14,21], including for ISAV [3,8]. The mAbs developed in the present study recognised Segment 3 of the ISA virus encoding the viral NP, as indicated by the bands recognised in western blotting at 66–71 kDa [22]. Seven of the mAbs were shown to be specific for ISAV in ELISA, but only six (mAbs 1, 3, 15, 32, 33 and 37) recognised all isolates from Scotland, Norway and Canada. Of these, mAbs 3 and 32 were chosen for the development of the Luminex (Bio-Plex) assay. These same mAbs have also been used to develop a lateral flow immunoassay for ISAV, the diagnostic sensitivity and specificity of which was reported by Caraguel *et al*. [15]. The anti-ISAV mAbs stained infected cells in IFAT and under confocal microscopy were seen to stain virus in the cytoplasm, the nucleus and the nucleolus when cells were examined 3 d.p.i. The nucleolar localization of NP has previously been seen in infected cells [23,24].

The Bio-Plex array method is most commonly used in human and veterinary medicine for detection of multiple analytes such as antibodies against pathogens [25–27], microbial nucleic acids [28] or bacteria and their toxins [29,30]. In addition, cytokine profiles can be quantified as a measure of vaccine efficacy as shown in a study using a Bio-Plex assay to measure swine cytokines following vaccination of pigs with Porcine reproductive and respiratory syndrome virus [31]. This technology has not as yet been applied for the detection of pathogens or immune response in aquaculture species; although a related method (Pepscan) using the Luminex platform has been applied in fish vaccine development with regards to epitope mapping for Beta nodavirus in sea bass [32].

The Bio-Plex assay developed here using mAbs 3 and 32 to detect ISAV in fish plasma is both sensitive and relatively rapid. The entire assay can be completed within 4 h and was as sensitive as the RT-qPCRs used to validate the Bio-Plex assay. Whilst not as fast as the run time of a RT-qPCR (80 min plus 1h extraction time [33]) further optimisation may be possible e.g. reducing the incubation time with plasma to 1h and the SA-PE incubation to 20 min would reduce the total run time to 160 min. In addition, there are advantages of the Bio-Plex assay over RT-qPCR in that non-lethal samples can be used (plasma), there is less risk of contamination and potentially multiple pathogens or immune responses can be assayed for simultaneously saving time and resources. The assay offers fast turn-around time when testing diagnostic samples, which is beneficial for early diagnosis of ISAV as this can significantly improve disease outcomes during ISA outbreaks [3]. All pathogenic strains of ISAV reported to date contain an HPR deletion with respect to the putatively ancestral, non-virulent HPR0 virus [34]. According to current EU legislation any detection of ISAV has to be followed by sequencing to confirm whether the detected ISAV is a HPRdel or HPR0 strain (COMMISSION IMPLEMENTING DECISION (EU) 2015/1554 of 11 September 2015). The Bio-Plex assay detects virus in plasma and the non-pathogenic variant, (ISAV-HPR0), replicates mainly in the gills causing only transient subclinical infection [34]. The EU directive also states that suspicion of ISAV must be corroborated by virus detection by two diagnostic methods with independent principles of detection, such as RT-qPCR and IHC. This study has provided an additional method of detection of the virus which could potentially replace more subjective methods such as IHC/IFAT.

The MFI of the detection antibody was obtained for 100 beads, which is equivalent to a single plasma sample being analysed one hundred times per analyte compared to only once in an ELISA reaction, which makes the Bio-Plex assay a more robust assay than an ELISA [26,35]. The assay was repeatable and precise as shown by low inter-assay variability i.e. a CV value of 7.6% and low intra-assay variability (CV of 9.1%).

The preferred methods currently advised by the OIE [3] to detect ISAV are isolation in cell culture with virus identification and RT-qPCR followed by sequencing. In order to compare the sensitivity of the Bio-Plex assay with the RT-qPCR method, an absolute RT-qPCR using existing primers for segment 8 [19] was performed according to the method previously described [17,18]. Absolute quantification involves construction of a standard curve using *in vitro* transcribed RNA standards as described here or using known copy numbers of plasmid DNA [17]. Absolute quantification of the virus by PCR is based on genomic viral RNA copy numbers present in the sample relative to a constant copy number [17]. The RT-qPCR platform based on SYBR^®^ Green real time chemistry or TaqMan^®^ chemistry, used in the current study, targeted Segment 8 of ISAV genome. The dynamic range of the TaqMan^®^ assay was 1.7 x 10^8^–25 copies/μl, the detection limit was 25 RNA copy numbers and was found to be more sensitive than the SYBR^®^ Green assay, confirming what Snow *et al*. [19] had previously reported for the two RT-qPCR chemistries.

Although the Bio-Plex assay detects Segment 3 of the virus (which encodes viral NP), and the RT-qPCR targets Segment 8 (which encodes the matrix protein of the virus), there was a strong relationship (correlation) between the detection of ISAV by the Bio-Plex assay and the two qPCRs, seen when measuring virus in both infected cell culture supernatant and infected plasma samples. This indicates the developed Bio-Plex assay is equally sensitive and specific for detection of ISAV when compared to RT-qPCR.

Detection of virus using this bead-based assay could be a simple cost effective way of screening non-lethal diagnostic samples compared to RT-qPCR, without the need for RNA extraction, or having to deal with problems associated with RNA contamination. The reduced sample volume needed for the Bio-Plex assay (50μl compared to 150 μl for RNA extraction) is an additional advantage of this platform. The main advantage of using Bio-Plex technology is its potential to detect multiple targets simultaneously. Co-infections commonly occur in aquaculture [36–38], highlighting the need for rapid, sensitive diagnostic methods to detect multiple targets. In such cases the Bio-Plex platform could be a useful tool for the detection of multiple pathogens in a single sample. The use of nucleic acid detection bead-based multiplex assays, whereby beads are coupled with a nucleic acid probe has been successfully used to detect multiple bacterial pathogens [30]. This method has also been used in viral genotyping assays, whereby 46 viral papilloma strains were identified using nucleic acid probes [39], and has also been used to monitor the distribution of virus types and for vaccine efficacy. Many multiplex assays for the measurement of cytokines have also been developed in recent years. The complex nature of the cytokine response following vaccination or infection makes it necessary to measure multiple cytokines at the same time to evaluate the immune response [35,40]. These studies highlight the potential applications of this platform in fish immunology and diagnostics. Future work will concentrate on further development of the assay reported here to encompass additional viral and bacterial pathogens occurring in the aquaculture environment.

## Conclusion

The present study describes the development and characterisation of mAbs, which allow specific detection of ISAV from different geographical regions. These mAbs were used to develop a novel diagnostic method for ISAV using bead-based technology. The results suggest that the developed Bio-Plex assay is a valid addition to RT-qPCR for the detection of ISAV, and significantly reduces sample volume required when compared with traditional methods such as ELISA and RT-qPCR.

## Supporting Information

S1 Fig Optimisation of capture antibody concentration by ELISA. aISAV MAb 3 and 32 were used to coat the plate at 5μg/ml and 10 μg/ml and viral supernatant from SHK-1 cells was diluted to make a standard curve.
(TIF)

S2 Fig Comparison of detection of ISAV in plasma of control or infected fish by ELISA and BioPlex assay. ELISA plate and Bio-Plex beads coated with aISA MAb3 at 10ug/ml. aISA 32-biotin used as capture at 20ug/ml. Cut-off = blank + 6X SD.
(TIF)
