# Supplementary figures and images for: Development, Characterisation and Application of Monoclonal Antibodies for the Detection and Quantification of Infectious Salmon Anaemia Virus in Plasma Samples Using Luminex Bead Array Technology
Source: PLoS One. 2016 Jul 19;11(7):e0159155. doi: 10.1371/journal.pone.0159155 (PMC4951118; doi:10.1371/journal.pone.0159155)

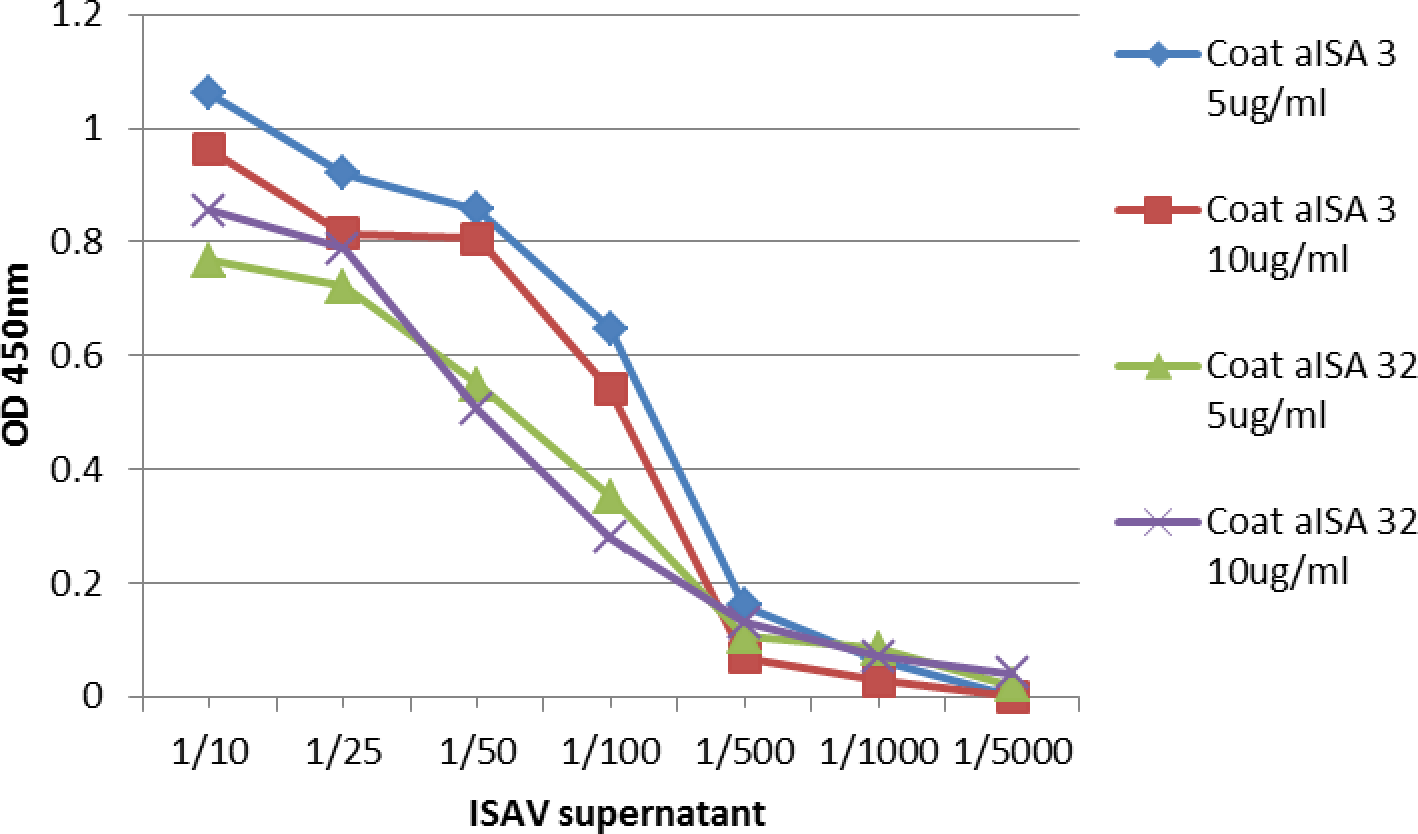

Supplement: S1 Fig — aISAV MAb 3 and 32 were used to coat the plate at 5μg/ml and 10 μg/ml and viral supernatant from SHK-1 cells was diluted to make a standard curve. (TIF) [file pone.0159155.s001.tif]

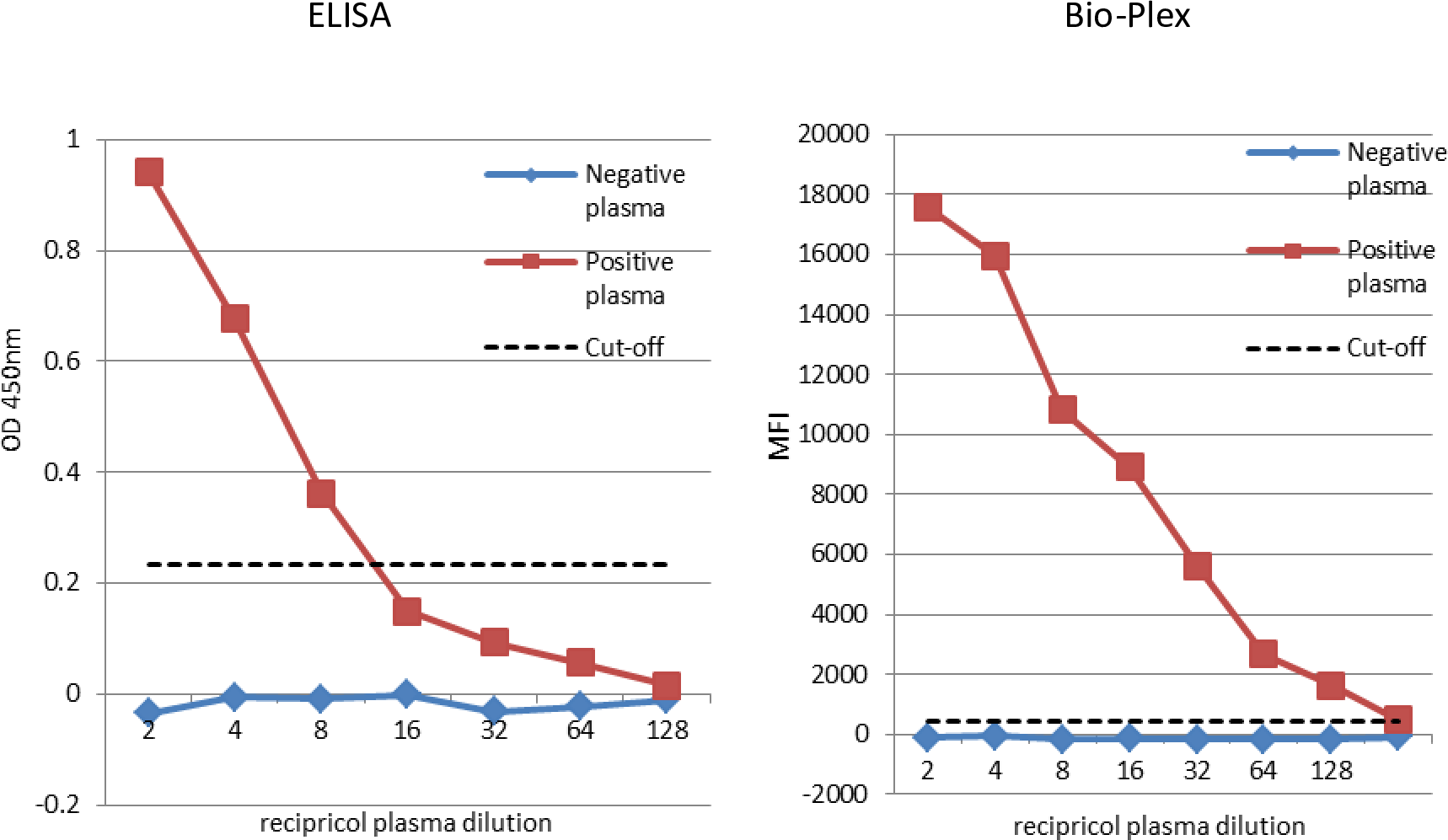

Supplement: S2 Fig — ELISA plate and Bio-Plex beads coated with aISA MAb3 at 10ug/ml. aISA 32-biotin used as capture at 20ug/ml. Cut-off = blank + 6X SD. (TIF) [file pone.0159155.s002.tif]
